# Margin-Based Modal Adaptive Learning for Visible-Infrared Person Re-Identification

**DOI:** 10.3390/s23031426

**Published:** 2023-01-27

**Authors:** Qianqian Zhao, Hanxiao Wu, Jianqing Zhu

**Affiliations:** 1 College of Information Science and Engineering, Huaqiao University, Xiamen 361021, China; 2 College of Engineering, Huaqiao University, Quanzhou 362021, China; 3 Xiamen Yealink Network Technology Company Limited, Xiamen 361015, China

**Keywords:** deep learning, maximum mean discrepancy, visible-infrared person re-identification

## Abstract

Visible-infrared person re-identification (VIPR) has great potential for intelligent transportation systems for constructing smart cities, but it is challenging to utilize due to the huge modal discrepancy between visible and infrared images. Although visible and infrared data can appear to be two domains, VIPR is not identical to domain adaptation as it can massively eliminate modal discrepancies. Because VIPR has complete identity information on both visible and infrared modalities, once the domain adaption is overemphasized, the discriminative appearance information on the visible and infrared domains would drain. For that, we propose a novel margin-based modal adaptive learning (MMAL) method for VIPR in this paper. On each domain, we apply triplet and label smoothing cross-entropy functions to learn appearance-discriminative features. Between the two domains, we design a simple yet effective marginal maximum mean discrepancy (M${}^{3}$D) loss function to avoid an excessive suppression of modal discrepancies to protect the features’ discriminative ability on each domain. As a result, our MMAL method could learn modal-invariant yet appearance-discriminative features for improving VIPR. The experimental results show that our MMAL method acquires state-of-the-art VIPR performance, e.g., on the RegDB dataset in the visible-to-infrared retrieval mode, the rank-1 accuracy is 93.24% and the mean average precision is 83.77%.

## 1. Introduction

**Figure 1:**
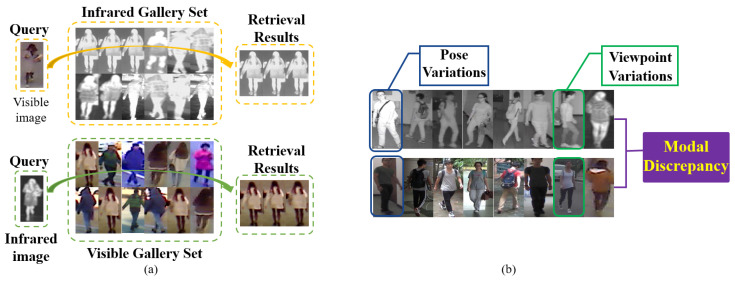
(**a**) The schematic diagram of visible-infrared person re-identification (VIPR) and (**b**) adverse factors in VIPR.

Visible-infrared person re-identification (VIPR) [1,2,3,4,5,6], as illustrated in Figure 1a, is important in smart city constructions because it can help find suspicious persons from massive traffic videos. As shown in Figure 1b, VIPR not only suffers from pose variations and viewpoint variations that commonly exist in traditional person re-identification [7,8,9,10] based on visible images but also encounters a huge modal discrepancy between visible and infrared images. The huge modal discrepancy is a serious challenge to VIPR because it leads to different identities of the same modality that are more similar than the same identity of different modalities. Therefore, how to to properly deal with modal discrepancies is crucial to VIPR.

To reduce the adverse effect of modal discrepancies, on the one hand, recent VIPR approaches [11,12,13,14,15] focus on adversarial-based modal conversion, which learns convert between visible and infrared data to ensure the two different modalities are uniform. For example, Wang et al. [16] applied a generative adversarial network (GAN) to produce fake infrared images from visible images and trained the VIPR model on both fake and real images to alleviate any modality discrepancies. Liu et al. [17] designed deep skip-connection generative adversarial networks to realize a high-quality cross-modal style translation to reduce modality discrepancies. Huang et al. [18] proposed using third modality data containing both visible and infrared information to prevent the information from the infrared modality from being overwhelmed during training. The third modality data were also produced by using a generative adversarial network. Choi et al. [19] proposed a hierarchical cross-modality disentanglement method to automatically disentangle the identity-discriminative factors and identity-excluded factors, creating a bridge between visible and infrared images to reduce modality discrepancies. Hu et al. [13] applied two modality-specific encoders and a modality-generic encoder to learn modality-specific and modality-generic features and then used two modality-specific decoders to generate synthetic images to compensate the missing modal data. We acknowledge that the adversarial way is effectively targeted to eliminate modal discrepancies, but it requires a list of complex sub-networks accompanied by the risk of model collapse and a high training computation cost.

On the other hand, domain adaptation [20,21,22,23,24,25] is a potential solution for VIPR because it has the goal of aligning distributions of source and target domains. The maximum mean discrepancy (MMD) [23,24,25] loss function is popular in domain adaptation and, recently, it has been applied to VIPR [26,27,28]. For example, Alehdaghi et al. [26] utilized the MMD loss function to determine the domain shift between visible and infrared modalities to provide additional information for training VIPR models. The Gram matrix-based MMD [27] method reduces modality variances in a latent space, learning modal-invariant features. The class-specific maximum mean discrepancy (CSMMD) loss function [28] independently reduces the modal discrepancies of visible and infrared images of each class. With the help of domain adaptation, those VIPR methods gain good performance, but there is still room for improving VIPR. Because VIPR is not a pure domain adaptation task, it has complete identity information on both visible and infrared modalities, so that it could not apply an unreserved maximum mean discrepancy suppression method to remove modal discrepancies, otherwise it would lose discriminative appearance information.

To this end, we propose a margin-based modal adaptive learning (MMAL) method for VIPR in this paper. In our MMAL method, we apply triplet and label smoothing cross-entropy functions to learn appearance-discriminative features and optimize maximum mean discrepancies to encourage the learned features to be modal invariant. Different from existing methods [27,28], our MMAL method does not focus on improving MMD to accurately measure the difference distribution of visible and infrared modalities, but pays attention to keeping a good balance of modal discrepancy suppression and appearance-discrimination learning. Thus, the main novelty of this paper is that our MMAL method designs a simple yet effective marginal strategy to avoid an excessive suppression of modal discrepancies to protect the features’ discriminative ability for boosting VIPR.

The contributions of this paper can be summarized as follows. (1) We design a margin-based modal adaptive learning (MMAL) method to join optimize modal discrepancies and discriminant appearances for VIPR, which could balance modal invariant and appearance discrimination via a marginal maximum mean discrepancy (M${}^{3}$D) loss function. (2) The experimental results on RegDB [29] and RGBNT [30] datasets demonstrate that our method acquires state-of-the-art performance, e.g., on the RegDB dataset, for the visible-to-infrared retrieval mode, the rank-1 accuracy is 93.24% and the mean average precision is 83.77%. The rest of this paper is organized as follows. Section 2 reviews the related work. Section 3 describes the proposed method in detail. Section 4 presents the experimental results to analyze our method’s superiority. Section 5 concludes this paper.

## 2. Related Work

In this section, we review the related works of this paper from two aspects: (1) recent VIPR progress and (2) domain adaption.

### 2.1. Recent VIPR Progress

**Figure 2:**
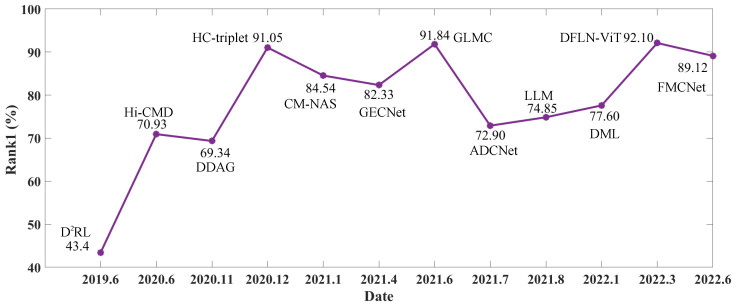
The Rank-1 (i.e., rank-1 accuracy) comparison of state-of-the-art works.

As shown in Figure 2, VIPR has been a hot topic in the past three years and has progressed remarkably. For example, the D${}^{2}$RL method [31] acquired a 43.4% rank-1 accuracy in 2019, the Hi-CMD method [19] gained a 70.93% rank-1 accuracy in 2020, and the DFLN-ViT [32] and FMCNet [33] methods obtained a 92.10% rank-1 accuracy and an 89.12% rank-1 accuracy, respectively, in 2022. The huge modal discrepancy of visible and infrared images are still a great challenge for VIPR; recent methods mainly focus on two aspects: (1) feature alignment and (2) adversarial conversion. A more detailed survey follows.

Regarding feature alignment, there are a lot of approaches [5,34,35,36,37,38,39,40,41,42,43,44,45,46,47]. The most popular architecture [5,35,48] is a double-stream deep network, where shallow layers are independent for learning modal-specific features and deep layers are shared for learning modal-common features. Some researchers improved the double-stream architecture via fine part alignment designs [40,49], attention mechanisms [35,36], or new neural structures, such as graph [27] and transformer [32,50].

In addition to architecture works, researchers also focus on designing alignment loss functions [39,44,45,46,47,51,52]. For example, Zhu et al. [51] designed a hetero-center loss function to constrain the intra-class center distance between two different modalities. Feng et al. [52] learned a center for each class and pulled the sample to the center of the same class as well as pushed the sample to the center of different classes; thus, the features of intra-class samples of different modalities were compacted with the center.

Compared to feature alignment, adversarial conversion [4,6,13,14,19,31,41,53] is newer on the whole. The main idea of adversarial conversion is to learn a conversion between visible and infrared data to ensure that different styles of visible and infrared images are uniform. For example, Wang et al. [31] converse the visible (or infrared) images to their infrared (visible, respectively) version, which are combined to form multi-spectral images for feature learning. Zhong et al. [53] proposed a gray-scale enhancement colorization network, which learns the correspondence between single-channel infrared images and three-channel visible images by generating intermediate gray-scale images as auxiliary information to colorize the single-modality infrared images. Furthermore, some disentanglement methods [13,14,19] specifically consider pedestrians’ appearance characteristics—gender or age information are modal insensitive but clothes colors are only for visible images—to generate synthetic images for reducing modal discrepancies.

In summary, both feature alignment and adversarial conversion methods have greatly improved VIPR, but VIPR is still far weaker than single-modal person re-identification due to the severe challenge of huge modal discrepancies between visible and infrared images.

### 2.2. Domain Adaption

Domain adaption aims to reduce the gap between source and target domains and eliminate the domain shift, so that the trained model could learn domain-invariant features, which has a lot of applications, such as unsupervised segmentation [54,55], unsupervised classification [24], and cross-modal action recognition [56]. In the domain adaption research field, the maximum mean discrepancy (MMD) [57,58] is commonly-used. Recently, MMD has been used in unsupervised person re-identification [59,60,61,62,63]. For example, Mekhazni et al. [59] proposed a dissimilarity-based maximum mean discrepancy loss function to align the pair-wise distance distributions between source and target domains. Yang et al. [62] explored the usability of MMD in learning multi-granularity domain-invariant features to overcome the sub-optimal alignment of global feature distributions.

We note that there are some MMD-based VIPR methods [26,27,28] highly related to our approach. The [26] method directly uses MMD, while [27,28] designs improved MMD to measure the distribution difference of visible and infrared modalities. No matter whether they are directly using MMD or improving MMD, those methods neglect an essential difference between domain adaptation and VIPR, that is, both two domains of VIPR have identity information and simultaneously require appearance discriminability. If the modal adaption is overemphasized from using MMD or improved MMD, the discriminative learning on visible and infrared modalities would be harmed because modal adaption pursuing modal distribution consistency is not always in line with the appearance discriminability on different modalities. Different from those MMD-based VIPR methods not considering the potential conflict of modal-invariant and appearance discrimination, our method pays attention to design a marginal strategy to avoid an excessive optimization of modal discrepancies, so that it protects its appearance-discriminative ability and acquires a good VIPR performance.

## 3. Methodology

In this section, we describe our method from two main aspects. (1) The margin-based modal adaptive learning (MMAL), which aims to learn modal-invariant yet appearance-discriminative features. (2) The deep network-based VIPR model, which explains how to use MMAL to supervise deep network learning features and how to adopt the learned features to realize VIPR.

### 3.1. Margin-Based Modal Adaptive Learning

The MAL consists of two types of loss functions, i.e., marginal maximum mean discrepancy (M${}^{3}$D) and appearance-discriminative loss functions. The former is responsible for modal-invariant and the later is in charge of appearance-discriminant functions.

#### 3.1.1. Marginal Maximum Mean Discrepancy Loss

Assume that a mini-batch consists of *X* and *Y*, which are two matrices carrying N×d features extracted from visible and infrared images, that is, $X=\left[x_{1},x_{2},\cdots ,x_{N}\right]\in R^{N\times d}$ and $Y=\left[y_{1},y_{2},\cdots ,y_{N}\right]\in R^{N\times d}$, where *N* is the number of visible or infrared samples, and *d* is the dimension of features extracted from images. The deep network for extracting features will be described later. The maximum mean discrepancy (MMD) loss function [57,58] is defined as follows:

(1) $$\begin{array}{c} \begin{array}{cc} L_{MMD}\left(X,Y\right) & ={∥\frac{1}{N}\sum_{i=1}^{N}\phi \left(x_{i}\right)-\frac{1}{N}\sum_{i=1}^{N}\phi \left(y_{i}\right)∥}_{H}^{2}\\ \; & =\frac{1}{N^{2}}\sum_{i=1}^{N}\sum_{j=1}^{N}\phi {\left(x_{i}\right)}^{⊤}\phi \left(x_{j}\right)-\frac{2}{N^{2}}\sum_{i=1}^{N}\sum_{j=1}^{N}\phi {\left(x_{i}\right)}^{⊤}\phi \left(y_{j}\right)+\frac{1}{N^{2}}\sum_{i=1}^{N}\sum_{j=1}^{N}\phi {\left(y_{i}\right)}^{⊤}\phi \left(y_{j}\right)\\ \; & =∥\frac{1}{N^{2}}\sum_{i=1}^{N}\sum_{j=1}^{N}K\left(x_{i},x_{j}\right)-\frac{2}{N^{2}}\sum_{i=1}^{N}\sum_{j=1}^{N}K\left(x_{i},y_{j}\right)+\frac{1}{N^{2}}\sum_{i=1}^{N}\sum_{j=1}^{N}K\left(y_{i},y_{j}\right)∥, \end{array} \end{array}$$

where ϕ(·) is a feature map function, if it is an identity function, the MMD loss function could simply compute the discrepancy between the samples’ means; H represents a reproducing kernel Hilbert space; $∥\cdot ∥$ is a norm calculation; and $K\left(x,y\right)$ is a kernel function, i.e., $K\left(x,y\right)=\langle \phi \left(x\right),\phi \left(y\right)\rangle$. In practice, the combination of multiple Gaussian kernels is a good choice for constructing a kernel function $K\left(x,y\right)$, as follows:

(2) $$K\left(x,y\right)=\sum_{i=1}^{L}\beta_{i}G_{i}\left(x,y\right),\beta_{l}\geq 0,\sum_{i=1}^{L}\beta_{i}=1,$$

where $G_{i}$ represents the *i*-th Gaussian kernel; $\beta_{i}$ is related to the variance of $G_{i}$, which is to guarantee that K is characteristic.

Considering that both the two domains of VIPR have supervisory information, VIPR could not completely pursue homogeneous features, otherwise, the features’ discriminative ability would be harmed. Therefore, we design a margin strategy to avoid an excessive reduction in modal discrepancies. The margin strategy is formulated as a marginal maximum mean discrepancy ($M^{3}D$) loss function in Equation (3).

(3) $$\begin{array}{c} L_{M^{3}D}=\max\left(L_{MMD}-\tau ,0\right) \end{array}$$

where τ>0 is a margin used to keep a boundary to avoid an excessive optimization of MMD. The default value of τ is 0.01.

#### 3.1.2. Appearance-Discriminative Loss

In this paper, we apply two types of appearance-discriminative loss functions, namely, the hard mining triplet (TRI) loss function [64] and the label-smoothing cross-entropy (LSCE) loss function [65]. The hard mining triplet (TRI) loss function is defined as:

(4) $$\begin{array}{c} L_{TRI}=\frac{1}{M}\sum_{i=1}^{M}\log\left[1+e^{\max_{f_{p}\in P_{i}}∥f_{i}-f_{p}{∥}_{2}-\min_{f_{n}\in N_{i}}{∥f_{i}-f_{n}∥}_{2}}\right], \end{array}$$

where M=2×C×K denotes the number of images in a mini-batch and *C* represents the number of classes and *K* denotes the number of visible images or infrared images of each class in the mini-batch; $f_{i}\in R^{d}$ is a *d*-dimensional feature corresponding the *i*-th image; and $P_{i}$ and $N_{i}$ denote the positive set and the negative set of the *i*-th image, respectively. Here, the positive set $P_{i}$ contains images of the same class to the *i*-th image and the negative set $N_{i}$ includes images of different classes from the *i*-th image.

The label-smoothing cross-entropy (LSCE) loss function is defined as follows:

(5) $$\begin{array}{c} L_{LSCE}=-\frac{1}{M}\sum_{m=1}^{M}\sum_{k=1}^{K}\epsilon_{m,k}log\left(p_{m,k}\right), \end{array}$$

where $p_{m,k}$ represents the posterior probability of the *m*-th image belonging to the *k*-th class, which is calculated using a softmax function; $\epsilon_{m,k}$ is a label-smoothing indicator function formulated as follows:

(6) $$\epsilon_{m,k}=\{\begin{array}{cc} 1-\frac{(K-1)\zeta }{K}, & x_{m}\in k-\mathrm{th}\mathrm{class},\\ \frac{\zeta }{K}, & x_{m}\notin k-\mathrm{th}\mathrm{class}, \end{array}$$

where ζ is a manual setting constant used to control the label-smoothing degree, which is usually set to 0.1 in practice.

### 3.2. Deep Network-Based VIPR

**Figure 3:**
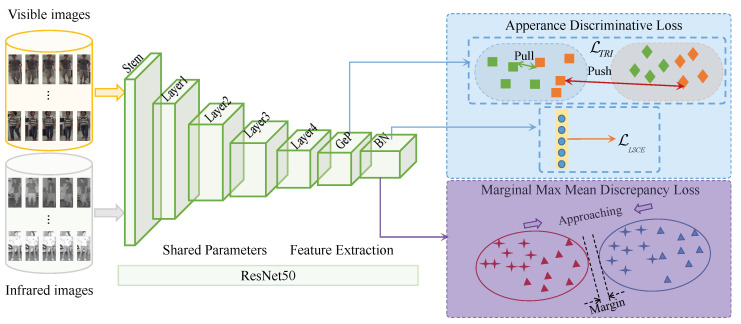
The visible-infrared person re-identification model via margin-based modal adaptive learning.

Following the existing VIPR works [35,38,41], we apply the popular residual network [66], namely, ResNet50, to construct a backbone for VIPR, as shown in Figure 3. Regarding the architecture, ResNet50 is a sequence with a stem layer (Stem), four residual groups (i.e., Layer-1–Layer-4), a generalized-mean pooling (GeP) [38] layer, and a batch normalization (BN) [64] layer. The Stem is a sequence of a 3×3 convolutional layer, a BN layer, a ReLU [67,68] layer, and a 2-stride max-pooling layer. Regarding the supervision, we assign the $L_{TRI}$ of Equation (4) on the GeP layer and set the $L_{LSCE}$ of Equation (5) and the M${}^{3}$D loss function $L_{M^{3}D}$ of Equation (3) on the BN layer following the GeP layer. The supervision is further formulated as follows.

(7) $$\begin{array}{c} L_{Total}=\lambda L_{MMAL}^{\mathrm{BN}}+L_{TRI}^{\mathrm{GeP}}+L_{LSCE}^{\mathrm{BN}}, \end{array}$$

where λ>0 is a hyper-parameter used to control the contribution of the M${}^{3}$D loss function, so that it keeps a good balance of modal-invariant and appearance-discriminative optimizations. $L_{MAL}^{BN}$ means the $L_{MAL}$ is applied to the BN layer following the GeP layer and both $L_{TRI}^{\mathrm{GeP}}$ and $L_{LSCE}^{\mathrm{BN}}$ have similar naming schemes. As a result, both modal-adaptive and appearance-discriminative loss functions are jointly applied to guide the ResNet50 to learn modal-invariant yet appearance-discriminative features for VIPR. In summary, the flowchart of the margin-based modal adaptive learning for VIPR is organized as Algorithm 1.
**Algorithm 1** Margin-based Modal Adaptive Learning for VIPR**Input:** A training dataset DB containing visible and infrared images and class labels, the number of training epochs *E*, and an initial deep network Net.**Output:** An updated deep network Net.1:**for***t* = 1:*E* **do**2:    **Sampling:** Randomly choosing a mini-batch from the training dataset DB and the mini-batch consists of *K* visible and *K* infrared images of *C* classes.3:    **Extraction:** Using the deep network Net to extract features from images of the mini-batch.4:    **Loss Calculation:** Using the total margin-based modal adaptive learning loss function (i.e., $L_{Total}$ in Equation (7)) to compute the loss on the mini-batch.5:    **Updating:** Using the mini-batch stochastic gradient descent optimizer to calculate the gradients of $L_{Total}$ to update the deep network Net.6:**end for**

In the testing process, the $\ell_{2}$ normalized features from the GeP and BN layers are fused for evaluating the VIPR performance. First, both query and gallery images are fed into the ResNet50 to acquire features. Second, based on the features, the distances among the query and gallery images are computed. Third, distances are sorted in ascending order to find the top-k gallery images similar to how the query images obtain retrieval results for VIPR.

## 4. Experiments

In this section, we evaluate our MMAL method and compare it with state-of-the-art approaches recently published in top conferences (e.g., CVPR, ICCV, and ECCV) or journals (e.g., IEEE T-CSVT, T-IP, T-MM, and T-NNLS). Two open datasets (RegDB: https://drive.google.com/file/d/1gnVt9GIQSvium_mcxc7AWLhSXm6lNWsa/view RGBNT201: https://doi.org/10.1609/aaai.v35i4.16467, mail to: ziwang1121@foxmail.com, accessed on 16 December 2022), namely, RegDB [29] and RGBNT201 [30], are applied to construct experiments.

### 4.1. Datasets

The RegDB [29] dataset includes 4120 pedestrian images of 412 classes and each class has five visible images and five infrared images. The evaluation protocol on RegDB is based on the average of ten trials and each trial randomly selected 206 classes of 2060 images as a training set and the non-overlapping rest as a testing set. Besides, there are two retrieval modes, i.e., visible-to-infrared (V2I) and infrared-to-visible (I2V). The V2I retrieval mode applies visible probes to search from a infrared gallery and the I2V retrieval mode is the opposite.

The RGBNT201 [30] dataset is a newly released three-modal (i.e., visible, infrared, and thermal) pedestrian image database. According to the data division of [30], the training subset consisting of 141 classes of 3280 visible images and 3280 infrared images and the testing set of 30 other classes of 836 visible images and 836 infrared images. However, different from [30], we only use visible and infrared images of each class for VIPR. Similar to the evaluation on the RegDB dataset, there are V2I and I2V retrieval modes. Regarding the V2I retrieval mode, the probe set is constructed by randomly selecting 10 visible images from each class of the testing set and the gallery set contains all the infrared images of the testing set. The I2V retrieval mode has the similar probe and gallery constructions but the modality configuration are opposite to the V2I retrieval mode. For both V2I and I2V retrieval modes, the average of ten for testing is reported as the final result.

### 4.2. Performance Metrics

Similar to existing works [5,36,38], the cumulative match characteristic (CMC) curve and the mean average precision (mAP) are applied to evaluate the VIPR performance, which are formulated as follows.

Assume that *K* is the number of gallery images; the indicator function is represented by Match and, if a query *q* correctly appears in the top-*n* retrieval results, Match(q,n) is equal to 1 and 0 otherwise. Then, the CMC is defined in Equation (8).

(8) $$\mathrm{CMC}\left(n\right)=\frac{1}{N}\sum_{q=1}^{N}\mathrm{Match}(q,n),$$

where CMC(1) is represented as Rank1, which represents a rank-1 accuracy. Compared to CMC, mAP is a more comprehensive performance metric, which takes both precision and recall into account. The definition of mAP is formulated in Equation (9).

(9) $$\mathrm{mAP}=\frac{1}{N}\sum_{q=1}^{N}\mathrm{AP}(q),$$

where AP(q) is the area under the precision-recall curve of the query *q*.

### 4.3. Experimental Conditions and System Configurations

As performed in [35,38], data augmentation is a sequence of 144 × 288 uniformly resizing, z-score normalizing, random cropping, random erasing [69], and horizontal flipping operations and the ImageNet [70] pre-trained Resnet50 is applied to initialize the backbone. The network optimizer is the stochastic gradient descent (SGD) [71]. Each mini-batch has four classes and each class contains five visible and five infrared images. The network’s weight decays are set to be 0.0005 and the momentums are set to be 0.9. There are 50 epochs for the training process. The learning rates are initialized to 0.001 and linearly warmed up [72] to 0.01 in the first 10 epochs. Then, the learning rates are kept at 0.01 from the 11st to the 30th epochs. At last, the learning rates are maintained at 0.001 from the 31st to the 50th epochs. The software tools are Pytorch 1.7, CUDA 11.1, and Python 3.8. The operation system is Ubuntu 18.04.5 LTS. The hardware device is a GeForce RTX 3090 GPU.

### 4.4. Results

The comparisons of our MMAL method and state-of-the-art approaches on the RegDB and RGBNT201 datasets are, respectively, listed in Table 1 and Table 2.

As compared in Table 1, our MMAL method achieves the best performance for both V2I and I2V retrieval modes. For the V2I retrieval mode, our MMAL method acquires 93.24% Rank1 and 83.77% mAP, which outperforms GLMC [40] with a 1.40% higher Rank1 and a 2.35% larger mAP and HC-Triplet [5] with a 2.19% higher Rank1 and a 0.49% larger mAP. In addition, compared with the two MMD-based methods, namely, CM-NAS [28] and ECGRAPH [27], our MMAL method outperforms CM-NAS [28] with an 8.70% higher Rank1 and a 3.45% larger mAP and outperforms ECGRAPH [27] with a 17.66% higher Rank1 and a 15.91% larger mAP. Compared with the disentangle method called ADCNet [13], our MMAL method achieves a 20.34% higher Rank1 and a 17.27% larger mAP. For the I2V retrieval mode, we find that, although our MMAL still wins the first place in terms of Rank1 and mAP, the advantage is relatively weaker compared to the V2I mode. We hypothesize that the reason is that we do not separate the optimizations of the visible-infrared and infrared-visible pairs.

Due to the RGBNT201 dataset being newly released and not for VIPR initially, there are few studies reporting their results. Under this background, we select several state-of-the-art approaches that have performed well on the RegDB dataset according to Table 1 as competitors to our MMAL method on the RGBNT201 dataset. The comparison results are shown in Table 2. We can observe that our MMAL method achieves the best performance for both V2I and I2V retrieval modes.

**Figure 4:**
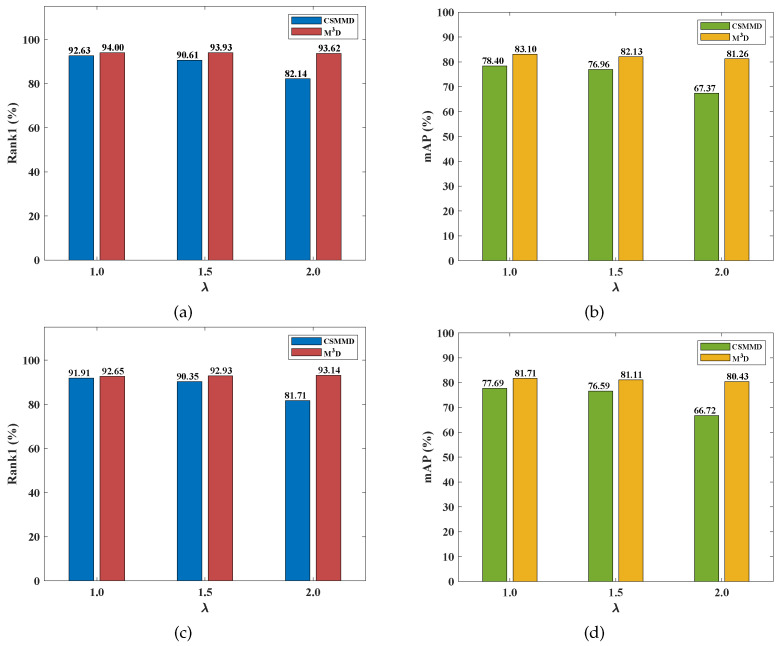
The comparison between our M${}^{3}$D and CSMMD [28] loss functions on the RegDB dataset. (**a**) V2I Rank1, (**b**) V2I mAP, (**c**) I2V Rank1, and (**d**) I2V mAP.

**Figure 5:**
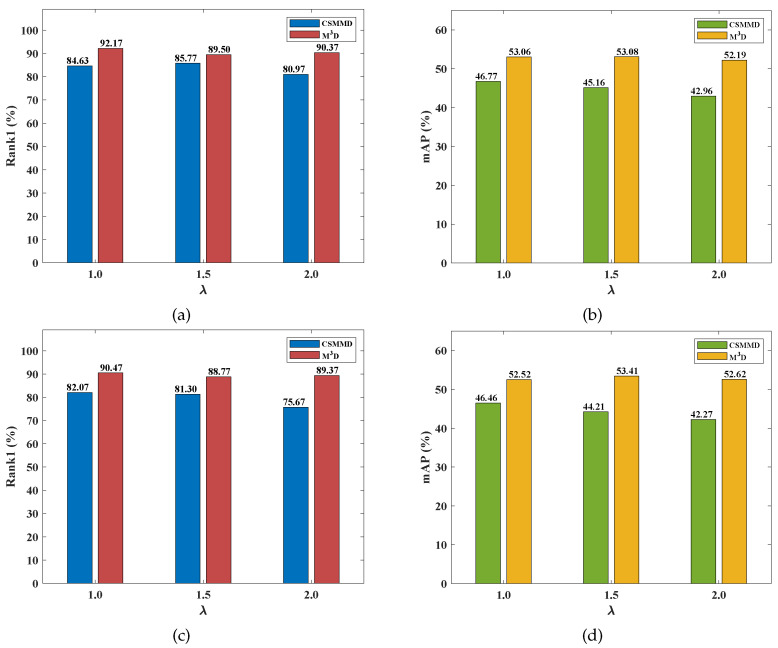
The comparison between our M${}^{3}$D and CSMMD [28] loss functions on the RGBNT201 dataset. (**a**) V2I Rank1, (**b**) V2I mAP, (**c**) I2V Rank1, and (**d**) I2V mAP.

To create a more concise comparison, we compared our M${}^{3}$D loss function and the class-specific max mean discrepancy (CSMMD) [28] loss function under the same conditions, i.e., using the same backbone and the same appearance-discriminative loss functions. The comparisons are shown in Figure 4 and Figure 5. We found that our M${}^{3}$D loss function consistently outperforms the CSMMD loss function for both V2I and I2V retrieval modes on the two datasets in terms of mAP and Rank1. For example, as shown in Figure 4a, λ is set to 1.0, 1.5, and 2.0, meaning our M${}^{3}$D beats the CSMMD by 1.37%, 3.32%, and 11.48% higher Rank1s, respectively. As shown in Figure 4b, when λ is set to 1.0, 1.5, and 2.0, our M${}^{3}$D is superior to the CSMMD by 4.70%, 5.17%, and 13.89% higher mAPs, respectively. We deduce that the disadvantage of CSMMD is because the class-specific modal discrepancy reduction more easily becomes redundant because of the intra-class distance optimization of the appearance-discriminative learning.

### 4.5. Analyses

In the above subsection, the performance advantage of our MMAL method has been validated via comparing two state-of-the-art methods. In what follows, we analyze our MMAL method’s advantage from three aspects: (1) The modal discrepancy suppression; (2) The role of the marginal strategy; (3) The analysis of the running time.

#### 4.5.1. Role of Modal Discrepancy Suppression

**Figure 6:**
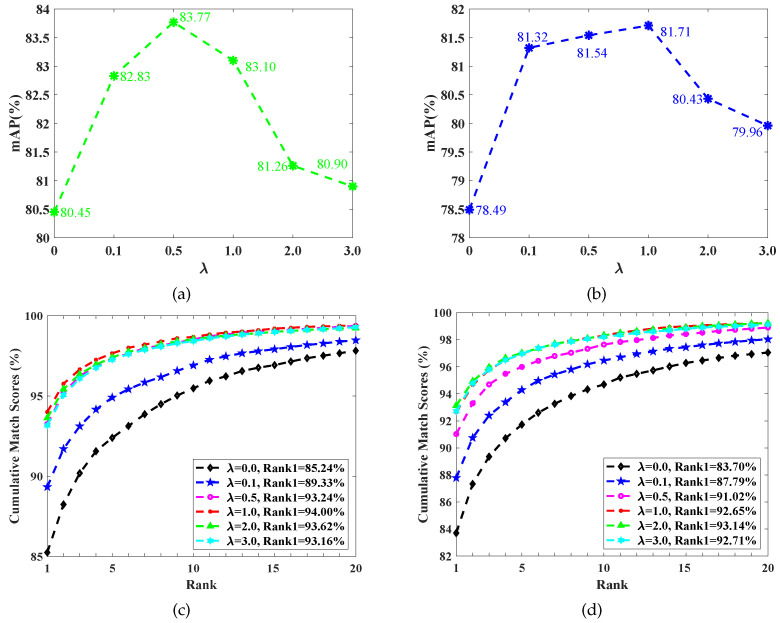
The performance of using different modal discrepancy restraining degrees (i.e., λ in Equation (7)) on the RegDB dataset. (**a**) V2I mAP, (**b**) I2V mAP, (**c**) V2I Rank1, and (**d**) I2V Rank1.

**Figure 7:**
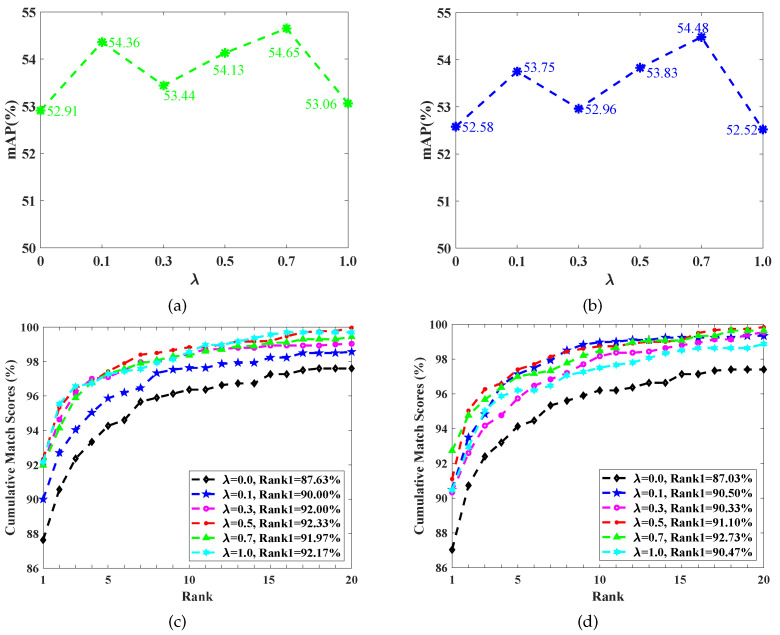
The performance of using different modal discrepancy suppression degrees (i.e., λ in Equation (7)) on the RGBNT201 dataset. (**a**) V2I mAP, (**b**) I2V mAP, (**c**) V2I Rank1, and (**d**) I2V Rank1.

Since the M${}^{3}$D loss function is responsible for the learning modal-invariant features and λ in Equation (7) controls the weight of the M${}^{3}$D loss function, we adjust the λ value to analyze the role of the modal discrepancy suppression. The results are shown in Figure 6 and Figure 7.

On the RegDB dataset, as shown in Figure 6, along with the increase in λ, mAP and Rank1 firstly improve and then deteriorate for both V2I and I2V retrieval modes. Given the V2I retrieval mode as an example, the optimal λ for mAP is 0.5, which provides the largest mAP, i.e., 83.77%, as shown in Figure 6a. Compared to the case of λ=0, the best performance improved the mAP by 3.32%. The best λ for Rank1 is 1.0, which leads to the highest Rank1, i.e., 94.00%, as shown in Figure 6c, compared to the case of λ=0, with the best performance improving the Rank1 by 8.76%. However, an overlarge λ causes performance degradation, such as the worst CMC curve from setting λ=3.0. A similar performance variation phenomenon occurs on the RGBNT201 dataset, as shown in Figure 7. These results suggest that an overemphasized modal discrepancy restraining would harm the features’ discriminant ability.

#### 4.5.2. The Role of Marginal Strategy

In this experiment, we fix λ to be 0.5 according the results presented in the subsection of analyzing the role of modal discrepancy suppression. As shown in Table 3, on the RGBNT201 dataset, most of the cases of the differently sized margins acquire improvements, compared to the naive case that does not apply any margin (i.e., τ=0). For example, the case of setting the margin to be 0.015 (i.e., τ=0.015) brings about 3% mAP improvements for both V2I and I2V retrieval modes. These results demonstrate that the marginal strategy avoiding the over-optimization of modal discrepancies to protect the features’ discriminant ability has a positive effect on improving VIPR.

#### 4.5.3. The Analysis of Running Time

**Figure 8:**
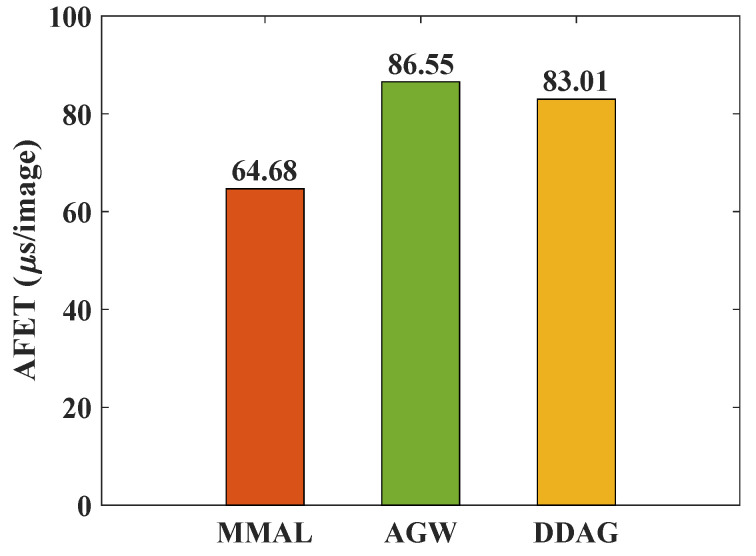
The running time performance of our MMAL approach and state-of-the-art methods. The batch size is set to 100 during testing.

Due to feature extraction costs of the higher inference times, following [76,77,78], we apply the average feature extraction time (AFET) per image as a running time indicator. The smaller the AFET per image, the better the running time performance would be. As shown in Figure 8, our MMAL method acquires the best AFET per each image performance. To be more specific, our MMAL method’s AFET per image is 21.87 μs smaller than that of the AGW [38] method and 18.33 μs smaller than that of the DDAG method [35]. The reason for this that both the AGW and DDAG methods apply a partial two-branch backbone, which is more complex than the single-branch backbone used in our MMAL method.

## 5. Conclusions

In this paper, we design a margin-based modal adaptive learning (MMAL) method for visible-infrared person re-identification (VIPR). VIPR is not completely the same as domain adaptation because VIPR has complete identity information on both visible and infrared modalities. Thus, VIPR requires a gentle domain adaptation that keeps a good balance of modal discrepancy suppression and appearance-discrimination learning. To this end, rather than directly using a traditional domain adaptation loss function, we design a simple yet effective marginal maximum mean discrepancy (M${}^{3}$D) loss function to avoid an excessive suppression of modal discrepancies to protect the features’ discriminative ability on both the visible and infrared modalities. Compared to the state-of-the-art methods, our method is competitive, e.g., on the RegDB dataset, for the visible-to-infrared retrieval mode, the rank-1 accuracy reaches 93.24% and the mean average precision reaches 83.77%. In addition, our experiments demonstrate that using our M${}^{3}$D loss function outperforms the naive case without using any domain adaptive loss function with a 4.70% higher rank-1 accuracy and outperforms the case using the traditional maximum mean discrepancy loss function with a 2.96% higher rank-1 accuracy on the RGBNT201 dataset.

## Figures and Tables

**Table 1:** The performance comparison of our MAL and state-of-the-art methods on the RegDB dataset. The V2I and I2V denotes the visible-to-infrared and infrared-to visible retrieval modes. The red, green and blue rows represents the 1st, 2nd and 3rd places, respectively.

Method	V2I		I2V		Reference
	Rank1 (%)	mAP (%)	Rank1 (%)	mAP (%)	
TSLFN+HC [51]	56.96	54.95	59.74	64.91	Neurocomputing 2020
X modality [68]	62.21	60.18	N/A	N/A	AAAI 2020
cm-SSFT [15]	65.4	65.6	63.8	64.2	CVPR 2020
DDAG [35]	69.34	63.46	68.06	61.80	ECCV 2020
Hi-CMD [19]	70.93	66.04	N/A	N/A	CVPR 2020
MACE [44]	72.37	69.09	72.12	68.57	TIP 2020
AGW [38]	70.05	66.37	N/A	N/A	TPAMI 2021
ADCNet [13]	72.9	66.5	72.4	65.3	ICME 2021
FBP-AL [41]	73.98	68.24	70.05	66.61	TNNLS 2021
LLM [52]	74.85	71.32	N/A	N/A	SPL 2021
ECGRAPH [27]	75.58	67.86	N/A	N/A	SPL 2021
MLCNN [73]	76.2	74.1	75.8	73.8	IEEE IOT 2021
SFANet [74]	76.31	68.00	70.15	63.77	TNNLS 2021
GECNet [53]	82.33	78.45	78.93	75.58	TCSVT 2021
MPANet [42]	83.7	80.9	82.8	80.7	CVPR 2021
CM-NAS [28]	84.54	80.32	82.57	78.31	ICCV 2021
MSA [75]	84.86	82.16	N/A	N/A	IJCAI 2021
HC-Triplet [5]	91.05	83.28	89.30	81.46	TMM 2021
GLMC [40]	91.84	81.42	91.12	81.06	TNNLS 2021
DMiR [43]	75.79	69.97	73.93	68.22	TCSVT 2022
DTRM [36]	79.09	70.09	78.02	69.56	TIFS 2022
MMAL	93.24	83.77	91.02	81.54	Ours

**Table 2:** The performance comparison of our MAL and state-of-the-art methods on the RGBNT201 dataset. The V2I and I2V denotes the visible-to-infrared and infrared-to visible retrieval modes.

Method	V2I		I2V		Reference
	Rank1 (%)	mAP (%)	Rank1 (%)	mAP (%)	
TSLFN+HC [51]	26.4	22.9	18.4	22.0	Neurocomputing 2020
DDAG [35]	73.5	45.5	73.35	45.8	ECCV 2020
CM-NAS [28]	75.3	43.3	75.6	45.3	ICCV 2021
AGW [38]	71.2	38.9	69.0	39.6	TPAMI 2022
DTRM [36]	82.0	44.5	83.9	45.1	TIFS 2022
MMAL	92.33	54.13	91.10	53.83	Ours

**Table 3:** The performance of using different sized margins on the M${}^{3}$D loss (i.e., Equation (3)) on the RGBNT201 dataset. The V2I and I2V denotes the visible-to-infrared and infrared-to visible retrieval modes.

τ	V2I		I2V	
	Rank1 (%)	mAP (%)	Rank1 (%)	mAP (%)
0	89.37	52.54	90.90	52.86
0.005	89.67	53.14	93.10	52.61
0.01	92.33	54.13	91.10	53.83
0.015	90.43	55.68	92.87	56.80

## Data Availability

Not applicable.

## Abbreviations

The following abbreviations are used in this manuscript:

VIPR	Visible-infrared person re-identification
GAN	Genaration adversarial network
MMAL	Margin-based modal adaptive learning
MMD	Max mean discrepancy
M${}^{3}$D	Margin max mean discrepancy
CSMMD	Class-specific maximum mean discrepancy
GeM	Generalized-mean pooling
BN	Batch normalization
Tri	Triplet
LSCE	Label-smoothing cross-entropy
BNNeck	Batch normalization neck
mAP	Mean average precision
CMC	Cumulative match characteristic
Rank1	rank-1 accuracy
SGD	Stochastic gradient descent
